# MicroLive: an image processing toolkit for quantifying live-cell single-molecule microscopy

**DOI:** 10.1093/bioadv/vbag095

**Published:** 2026-03-30

**Authors:** Luis U Aguilera, William S Raymond, Rhiannon M Sears, Nathan L Nowling, Brian Munsky, Ning Zhao

**Affiliations:** Department of Biochemistry and Molecular Genetics, University of Colorado-Anschutz Medical Campus, Aurora, CO, 80045, United States; School of Biomedical and Chemical Engineering, Colorado State University, Fort Collins, CO, 80523, United States; Department of Biochemistry and Molecular Genetics, University of Colorado-Anschutz Medical Campus, Aurora, CO, 80045, United States; Department of Biochemistry and Molecular Genetics, University of Colorado-Anschutz Medical Campus, Aurora, CO, 80045, United States; School of Biomedical and Chemical Engineering, Colorado State University, Fort Collins, CO, 80523, United States; Department of Biochemistry and Molecular Genetics, University of Colorado-Anschutz Medical Campus, Aurora, CO, 80045, United States

## Abstract

**Motivation:**

Advances in live-cell fluorescence microscopy have enabled us to visualize single molecules (such as mRNAs and nascent proteins) in real time with high spatiotemporal resolution. However, these experiments generate large datasets that require complex computational processing pipelines to derive meaningful and quantitative information, which is a technical barrier for many researchers.

**Results:**

Here, we introduce MicroLive, an open-source Python-based application for quantifying live-cell microscopy images. MicroLive provides an interactive Graphical User Interface (GUI) to perform key tasks, including cell segmentation, photobleaching correction, single-particle detection/tracking, spot intensity quantification, inter-channel colocalization, and time-series correlation analysis. As a ground-truth testing dataset, we used synthetic live-cell imaging data generated with the rSNAPed toolkit, demonstrating accurate extraction of biologically relevant parameters. Microscopy images of U-2 OS cells expressing a gene construct smHA-KDM5B-BoxB-MS2 were used to demonstrate the use of this software.

**Availability and implementation:**

MicroLive is distributed under a GPLv3 license and available on GitHub https://github.com/ningzhaoAnschutz/microlive. It can be installed via pip: pip install microlive.

## 1 Introduction

**Figure 1:**
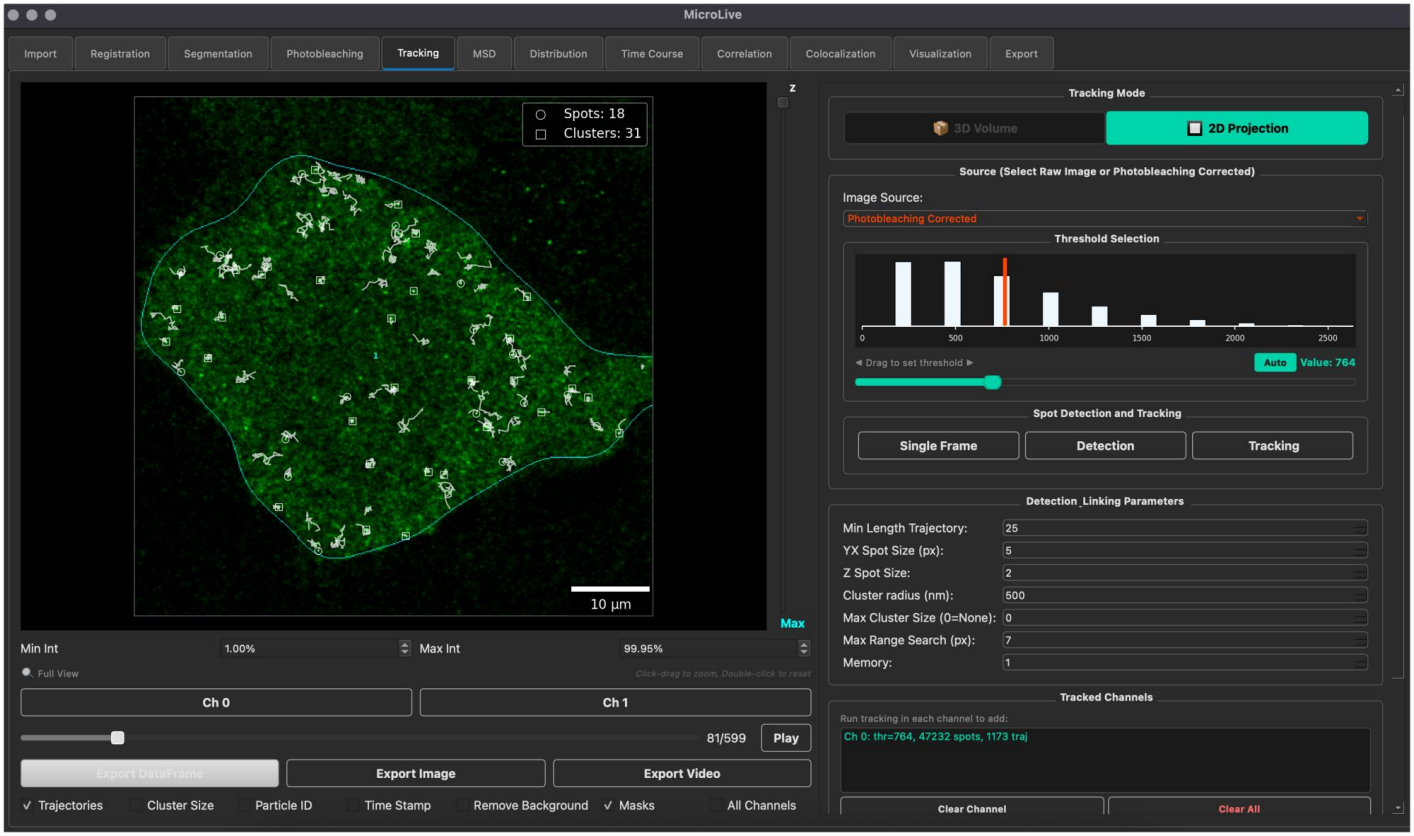
A snapshot of MicroLive. Multiple tabs allow users to perform cell segmentation, spot detection, particle tracking, intensity calculation, colocalization, and correlation analysis. The processed image represents a U-2 OS cell expressing the smHA-KDM5B-BoxB-MS2 gene construct.

Live-cell single-molecule microscopy is transforming our understanding of gene expression (Morisaki *et al.* 2024). Novel imaging strategies combine advanced live-cell fluorescence microscopy and genetically encoded fluorescent tagging systems, such as intrabody systems [e.g. SunTag (Tanenbaum *et al.* 2014, Yan *et al.* 2016) and frankenbodies (Zhao *et al.* 2019, Liu *et al.* 2021)] and RNA stem-loop systems [e.g. MS2 (Bertrand *et al.* 1998) and PP7 stem loops (Chao *et al.* 2008)], allowing researchers to visualize nascent proteins and mRNAs directly in real time in live cells (Sears *et al.* 2025). These technological advances have revealed complex phenomena, such as single mRNA translation (Yan *et al.* 2016, Morisaki *et al.* 2016, Wang *et al.* 2016, Wu *et al.* 2016, Pichon *et al.* 2016), translation bursts (Livingston *et al.* 2023), frameshifting (Lyon *et al.* 2019), and IRES-initiated translation (Koch *et al.* 2020). However, extracting meaningful information from live-cell microscopy is a complicated multi-step process, including cell segmentation, diffraction-limited fluorescent spots detection, linking detected spots across frames, quantifying spot intensity, and determining colocalization or temporal correlations between imaging channels (Khuperkar *et al.* 2020). Performing these analyses often requires a combination of software tools [e.g. ImageJ (Schindelin *et al.* 2012)] and custom scripts, which creates a technical barrier for many researchers. To address this, we introduce MicroLive, a user-friendly Graphical User Interface (GUI) platform for live-cell single-molecule imaging analysis. While our primary validation focuses on translation imaging, MicroLive is designed as a general-purpose toolkit applicable to diverse experimental scenarios, including transcriptional imaging, protein diffusion studies, and multicellular samples (Forero-Quintero *et al.* 2021, Vo *et al.* 2023). The main advantage of MicroLive is its ability to enable users to load multi-dimensional images and implement a complete image-processing pipeline through a point-and-click graphical interface, as shown in Fig. 1.

## 2 Method and implementation

MicroLive is implemented in Python with a modular architecture: the core image processing routines are coded as classes and reside in microlive/microscopy.py, while the code generating the GUI is located in microlive/gui/app.py. MicroLive consolidates commonly used image processing tasks into a single accessible platform, eliminating the need to chain together multiple independent tools (see Supplementary Information, available as supplementary data at *Bioinformatics Advances* online, for a comparison with existing software). MicroLive applies parallel computing to accelerate compute-intensive tasks, such as processing multiple-frame images. Additionally, MicroLive loads on-demand individual 2D frames or 3D stacks from the disk only when the frame or stack is displayed or processed, then frees the memory immediately. This on-demand strategy minimizes memory usage and supports large datasets.

MicroLive supports standard microscopy files like multi-dimensional TIFF or LIF. For unstructured TIFF files, a Jupyter Notebook is provided to convert them into the standard format used in MicroLive for downstream image analyses. Once loaded in MicroLive, microscopy images are internally converted into uint16 NumPy arrays. As a pre-processing step, the user can correct the loss of fluorescence intensity due to photobleaching (Miura 2020). Segmentation is supported through multiple methods: (i) Cellpose (Stringer *et al.* 2021), a deep learning approach that can perform cytosol and/or nucleus segmentation with support for time-varying masks; (ii) a watershed method for threshold-based segmentation (Vincent and Soille 1991); (iii) a manual outline tool that allows users to draw Regions of Interest (ROIs); and (iv) import of pre-computed masks generated by external tools. Masks are overlaid on images for validation and used to map detected spots to individual cells.

Particles or spots are detected using the TrackPy library (Allan *et al.* 2025) for 2D images or Big-FISH (Imbert *et al.* 2022) for 3D images. Automated threshold detection is available using a hybrid approach that combines methods from Big-FISH (Imbert *et al.* 2022) and TrueSpot (Hospelhorn *et al.* 2025) for robust threshold selection across diverse imaging conditions. Detected particles can be labeled as clusters if their size is multiple times larger than the user-defined particle size. Users can filter out large clusters that may represent aggregates. Particle trajectories are constructed using a nearest-neighbor algorithm with tunable displacement and memory parameters. Particle linking is supported for both 2D and 3D images. Importantly, MicroLive supports multi-channel tracking, allowing users to independently track spots in different channels using channel-specific detection parameters. The code offers visualization of the linked trajectories and the particle identifier, allowing users to detect and correct tracking errors in real-time. For multi-color images, MicroLive allows automated detection of colocalized spots by using a Convolutional Neural Network (CNN) (Newby *et al.* 2018) trained on manually-annotated images combined with augmented data to predict the presence of particles in a given ROI, or by using distance-based spatial proximity thresholds between tracked spots in different channels. The CNN architecture and performance metrics are provided in Fig. 2 and Table 1, available as supplementary data at *Bioinformatics Advances* online, respectively. Automated colocalization can be manually curated to reduce false positives and false negatives. Fluorescence intensities for the detected spots can be extracted over time using multiple methods, including total intensity, Gaussian fits, and background subtraction methods. MicroLive computes auto- and cross-correlation functions to reveal kinetic parameters such as dwell times and inter-signal time delays (Coulon and Larson 2016). All data generated by MicroLive can easily be exported as CSV files for downstream processing. Metadata containing all the parameters and thresholds used during the image processing steps is automatically generated and exported to ensure reproducibility. A complete description of the methods used in MicroLive is provided in the Supplementary File, available as supplementary data at *Bioinformatics Advances* online.

## 3 Validation and results

To verify our code with a ground truth dataset, we used synthetic movies generated with rSNAPed (Raymond *et al.* 2023). For this, a synthetic dataset containing 360 frames with a 5-s frame interval, two color channels, 512 × 512 pixels, and 80 mRNA spots was generated to model mRNA translation using an initiation rate of 0.04 1/sec and an elongation rate of 5 aa/sec. Photobleaching was simulated using a decreasing exponential function with a decay rate of 0.001 1/sec. Ground-truth positions and intensities were retained for benchmarking, and recovered results are provided in Fig. 3, available as supplementary data at *Bioinformatics Advances* online, showing a strong agreement between the tracked particles and the values used for the simulation. For example, MicroLive accurately detected an average of 66 mRNA spots at all time points. MicroLive estimated an intensity decay of 0.001 1/sec. Additionally, to determine whether MicroLive can correctly extract temporal intensity from the images, we calculated the autocorrelation function of nascent protein intensity traces and extracted initiation and elongation rates as described by Larson *et al.* (2011). We obtained a de-correlation time of ≈380 s, corresponding to an elongation rate of 5.0 aa/sec, and a value for the autocorrelation function at lag zero G(0)=0.08, corresponding to an initiation rate of 0.032 1/sec. A side-by-side comparison between synthetic data generated with the rSNAPed library and MicroLive recovered values is given in the Table 2, available as supplementary data at *Bioinformatics Advances* online.

To test MicroLive, we analyzed microscopy translation images of a gene construct smHA-KDM5B-BoxB-MS2 (Galindo *et al.* 2025) in live U-2 OS cells. The smHA-KDM5B-BoxB-MS2 (plasmid sequence is provided) construct consists of an N-terminal spaghetti monster HA (smHA) tag (Viswanathan *et al.* 2015) (including 10× HA tags) fused to our protein-of-interest KDM5B, 15× BoxB (Schärpf *et al.* 2000, Cialek *et al.* 2022, Galindo *et al.* 2025), and 24× MS2 stem-loops (Bertrand *et al.* 1998) in the 3′ untranslated region (UTR). Translation spots were identified by colocalized nascent protein spots visualized by anti-HA-frankenbody-HaloTag (Zhao *et al.* 2019) stained with JF646 dyes and mRNA spots labeled by tandem MS2 Coat Protein (tdMCP) (Bertrand *et al.* 1998) fused with tandem monomeric StayGold (tdmSG) (Hirano *et al.* 2022, Ando *et al.* 2024). The mRNA spots were tethered to the plasma membrane through the interaction between λN-CAAX and the BoxB stem-loops (Schärpf *et al.* 2000). The translation images were collected using a Leica Stellaris 5 confocal microscope with a 63× oil objective for 600 frames at a 1 frame per second (fps) rate with a single *z* plane. Images were loaded into MicroLive to perform cell segmentation, spot detection, intensity calculation, and correlation analyses. The complete quantification is presented in the Supplementary File, available as supplementary data at *Bioinformatics Advances* online.

To further demonstrate the versatility of MicroLive across a broad range of experimental scenarios, we provide additional implementations using published datasets: (i) multicellular segmentation and spot detection using data from Vo *et al.* (2023); (ii) protein diffusion analysis with Mean Squared Displacement (MSD) calculations using data from Zhao *et al.* (2019); and (iii) transcriptional imaging using MS2-labeled nascent RNA data from Forero-Quintero *et al.* (2021). Complete analyses are provided as Jupyter Notebooks in notebooks/examples/.

## 4 Conclusion

MicroLive is a toolbox to quantify single-molecule microscopy images. MicroLive unifies commonly used image processing tasks such as cell segmentation, spot detection, particle tracking, colocalization, and correlation analyses within a single accessible platform. The user-friendly GUI platform lowers the barrier for non-programmers to perform complex image-processing tasks. MicroLive is open-source with a GPLv3 license.

## Supplementary Material

vbag095_Supplementary_Data

## Data Availability

Source code is available in GitHub, at https://github.com/ningzhaoAnschutz/microlive. The data underlying this article are available in Zenodo, at https://dx.doi.org/10.5281/zenodo.19444594.
