# Supplementary material for: MicroLive: an image processing toolkit for quantifying live-cell single-molecule microscopy
Source: Bioinform Adv. 2026 Mar 30;6(1):vbag095. doi: 10.1093/bioadv/vbag095 (PMC13080936; doi:10.1093/bioadv/vbag095)
Supplement: vbag095_Supplementary_Data [file vbag095_supplementary_data.zip › 07-Apr-2026_110445_supplementary_information.pdf]

Supplementary Information for MicroLive: An Image Processing Toolkit for Quantifying Live-cell Single-Molecule Microscopy

Luis U. Aguilera[1], William S. Raymond[2], Rhiannon M. Sears[1], Nathan L. Nowling[1], Brian Munsky[2], and Ning Zhao[1]

[1]Department of Biochemistry and Molecular Genetics, University of Colorado-Anschutz Medical Campus, 80045, CO, USA

[2]School of Biomedical and Chemical Engineering, Colorado State University, 80523, CO, USA

General Architecture

MicroLive is a Python library with a Graphical User Interface (GUI) for quantitative analysis of live-cell single-molecule microscopy images. As illustrated in Figure S1, MicroLive allows the user to implement major tasks such as: i) data import and export, ii) preprocessing for registration, segmentation, and photobleaching correction, iii) spot detection for particle tracking, time course, and colocalization analyses, iv) statistical analyses for distributions and correlation calculations, and v) visualization for displaying crops and trajectories.

Image Loading

Images can be imported into the MicroLive via the **Open File** button located in the **Import** tab. MicroLive supports standard microscopy files, such as multi-dimensional TIFF/OME-TIFF and LIF. Once loaded, MicroLive automatically extracts critical metadata, including spatial dimension, pixel dimension, frame time intervals, laser intensities, and channel configuration. If any critical metadata is missing, MicroLive prompts the user to input values manually to ensure accurate unit conversion. For unstructured TIFF images, a Jupyter Notebook (`notebooks/microlive_converter.ipynb`) is provided to convert these images into the standard format. Once images are loaded into MicroLive, they are mapped into a standard five-dimensional array format (time, z, y, x, channel), ensuring consistent downstream processing. This process ensures that subsequent quantitative analyses, such as spot intensity measurement and particle tracking, are correctly performed and interpreted in terms of physical units and axis dimensions.

Cell Segmentation

To define cellular Regions of Interest (ROIs) for analysis, MicroLive supports both manual and automated segmentation methods. For manual segmentation, users can define ROIs by drawing directly on images using a built-in interactive polygon drawing tool. For automated segmentation, two approaches are available: (i) a watershed-based algorithm with a user-adjustable slider to define the segmentation threshold (Vincent and Soille, 1991), and (ii) deep learning-based segmentation using Cellpose (Stringer et al., 2021), which provides cytosol and/or nucleus segmentation with support for time-varying masks, and multi-cell segmentation. Each segmentation component (Cytosol and Nucleus) includes an independent size adjust slider (-20 to $+20$ px) for expanding or shrinking masks post-segmentation. Additionally, users can import pre-computed masks generated by external tools. Optionally, maximum temporal projections of the movies can be used as input for segmentation, which accounts for cell movement over time. The user-defined ROIs are accessible for all downstream analyses.

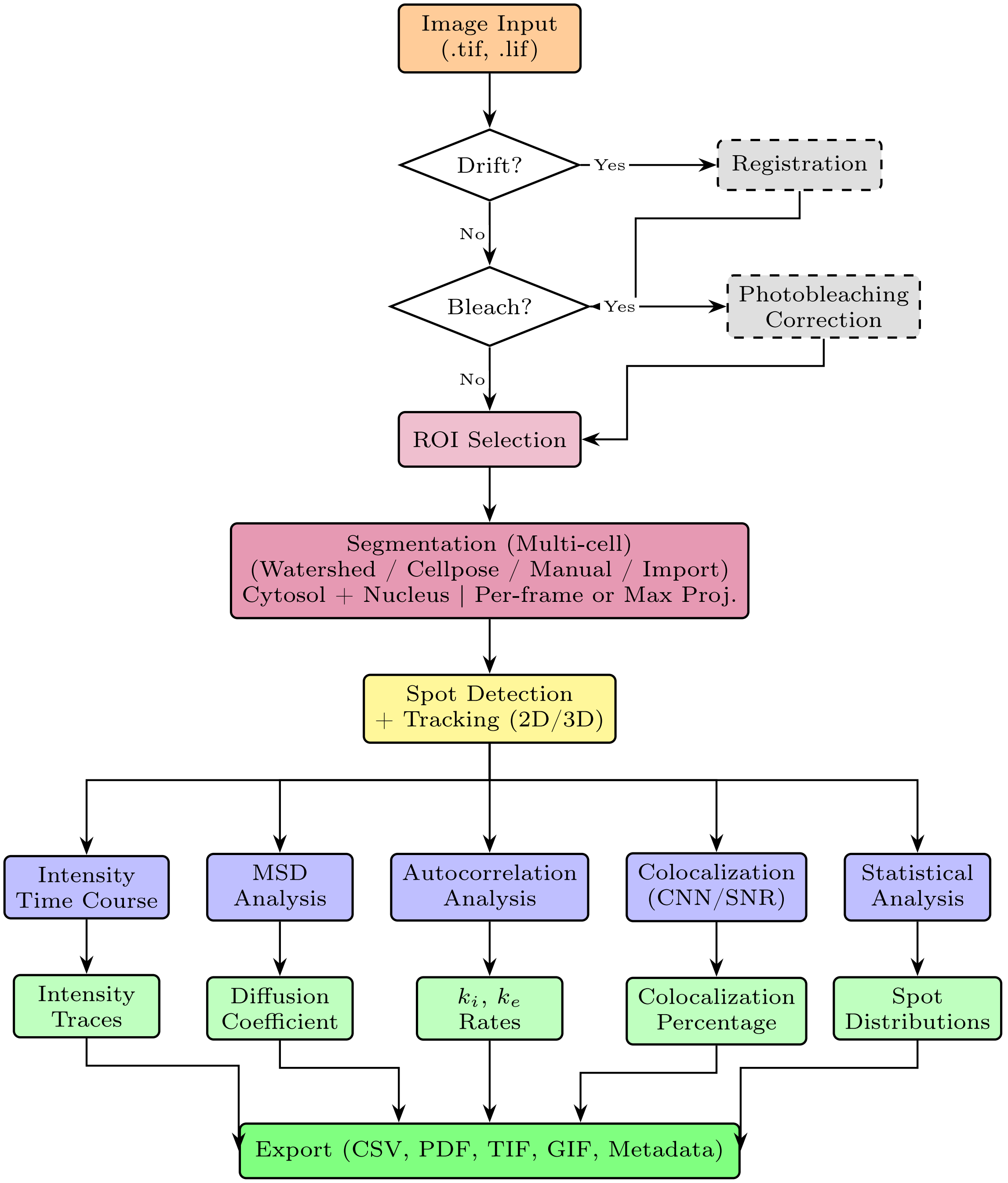

Figure S1: **MicroLive Workflow:** Image processing pipeline from input to final outputs. Dashed boxes indicate optional preprocessing steps. Segmentation supports four methods: Watershed (threshold), Cellpose (deep learning), Manual (polygon drawing), and Import (external masks). The pipeline supports both 2D and 3D tracking, with multiple downstream analysis modules including MSD analysis (diffusion coefficients), autocorrelation (transcription/translation rates), CNN-based colocalization, intensity time courses, and statistical analysis.

Photobleaching Correction

The **Photobleaching** tab corrects for progressive fluorescence decay by fitting an exponential model to the mean intensity time course and then rescaling each frame based on the fitted decay curve (Miura, 2020). The detailed method is shown as follows:

$$I_{\text{fit}}(t) = I_0\, e^{-kt}, \tag{1}$$

here, $I_{\text{fit}}(t)$ is the fitted fluorescence intensity at time t, I_0 is the initial intensity, and k is the decay constant.

To correct the raw images for photobleaching in channel c, we used a unit-normalized approach that approximates the intensity at $t = 0$ to remove the global photobleaching in all subsequent frames. Specifically, we introduce the following correction factor applied to each frame:

$$f^{(c)}(t) = \frac{I_{\text{fit}}^{(c)}(0)}{I_{\text{fit}}^{(c)}(t)}, \tag{2}$$

then each raw image at time t in channel c is multiplied by the correction factor, effectively restoring its intensity to the estimated unbleached level, as shown below,

$$I_{\text{corr}}^{(c)}(t) \;=\; f^{(c)}(t)\, I_{\text{raw}}^{(c)}(t). \tag{3}$$

Spot Properties

Spot Intensity

MicroLive provides three methods for quantifying spot intensities.

- The first method (Disk-Doughnut) uses local background subtraction (Lyon et al., 2019). For this, the mean intensities of the spot region D and an annular region R around the spot (local background) are determined, and then the local background intensity is subtracted from the spot intensity. That is:

$$I_{\text{spot}} = \frac{1}{s_{\text{spot}}^2} \sum_{(x,y)\in D} I(x,y) - \frac{1}{s_{\text{bg}}^2 - s_{\text{spot}}^2} \sum_{(x,y)\in R} I(x,y), \tag{4}$$

  where s_{spot} is the user-defined spot size in pixels, and s_{bg} is defined as an outer region extending 3 pixels beyond s_{spot} in each xy direction.

- For the second method, the spot intensity is estimated by fitting a 2D Gaussian function to determine its peak amplitude I_0 above the background. The intensity profile of a spot is modeled as

$$I_{\text{spot}}(x,y) = I_{\text{bg}} + I_0\, \exp\!\Bigg(-\tfrac{1}{2}\Big[\frac{(x-x_0)^2}{\sigma_x^2} + \frac{(y-y_0)^2}{\sigma_y^2}\Big]\Bigg), \tag{5}$$

  where I_{bg} is the local background level; σ_x, σ_y are the fitted spot widths along the x and y axes, respectively; I_0 is the peak amplitude of the Gaussian function; x_0 and y_0 are the coordinates of the center of mass of the detected spot.

- The third method calculates the integrated intensity by summing the pixel intensities for all pixels within the spot region D:

$$I_{\text{spot}} = \sum_{(x,y)\in D} I(x,y). \tag{6}$$

By default, the three methods are applied automatically by MicroLive, and their results are exported into the final dataframe.

Spot Size

For each detected spot, MicroLive first fits its fluorescence profile to a 2D Gaussian function (see Eq. 5). The spot size is then reported as the Full Width at Half Maximum (FWHM), defined as:

$$\mathrm{FWHM} = 2\sqrt{2\ln 2} \times \sigma_{xy}, \approx 2.355 \times \sigma_{xy}, \tag{7}$$

where σ_{xy} represents the mean of σ_x and σ_y obtained from Eq. 5.

For 3D particle tracking, in cases where large spots are detected (e.g., clusters), MicroLive computes a cluster size metric using the Big-FISH library (Imbert et al., 2022). This metric represents the number of individual spot detections grouped within a defined area. Identified clusters can be optionally excluded from downstream analyses.

Signal-to-Noise Ratio

MicroLive provides two methods for calculating the Signal-to-Noise Ratio (SNR) of detected spots, selectable via the `snr_method` parameter in the `Intensity` class.

SNR using the **Maximum Intensity (Default)** uses the maximum pixel intensity within the spot region as the signal (Imbert et al., 2022):

$$\mathrm{SNR}_{\mathrm{peak}} = \frac{I_{\mathrm{max}} - \mu_{\mathrm{bg}}}{\sigma_{\mathrm{bg}}}, \tag{8}$$

where I_{max} is the maximum pixel intensity within the spot region D, μ_{bg} is the mean background intensity in the annular region R, and σ_{bg} is the standard deviation of the pixel intensity in the region R.

SNR using the **Disk-Doughnut** uses the mean disk intensity (calculated using Eq. 4) as the signal:

$$\mathrm{SNR}_{\mathrm{disk}} = \frac{I_{\mathrm{spot}}}{\sigma_{\mathrm{bg}}}. \tag{9}$$

This method is more robust when imaging data is very noisy or spots are dim, where the maximum pixel value may be unreliable due to noise spikes.

Particle Detection and Tracking

The tracking module enables automated detection and linking of particles in either two or three dimensions. There are two main functionality modes: **Detection** and **Tracking**. In the **Detection** mode, MicroLive detects all particles in each frame, but no particle linking is applied. The **Tracking** mode implements both particle detection and particle linking. In 2D tracking (the default), z planes in each movie are maximum projected, and the TrackPy library (Allan et al., 2025) is used for spot detection and frame-to-frame linking of particle trajectories. In 3D tracking, MicroLive employs the Big-FISH library (Imbert et al., 2022) to detect fluorescent spots in 3D (across all z planes) and applies TrackPy for linking trajectories in x–y–z space over time. Importantly, MicroLive supports multi-channel tracking, allowing users to independently track spots in different channels using channel-specific detection thresholds and parameters. MicroLive also provides automated threshold detection for spot detection using a hybrid approach that combines methods from Big-FISH (Imbert et al., 2022) and TrueSpot (Hospelhorn et al., 2025). Users can click the "Auto" button to calculate and apply the optimal threshold.

Time Courses

Time course analysis can be used to track the temporal evolution of the image properties. For each frame, the number of detected spots in an ROI can be counted to produce a particle count vs. time curve. In addition, average intensity, SNR, spot size, and spot intensity values for all spots in the ROI can be plotted over time. All measurements can be plotted after photobleaching correction to ensure that observed temporal changes reflect true biological dynamics rather than gradual intensity loss by bleaching.

Spot Colocalization

When analyzing multi-channel (multi-color) fluorescence images, MicroLive enables users to detect spots in a reference channel and assess their colocalization with spots in a test channel. MicroLive provides four approaches to determine spot colocalization: (i) machine learning, (ii) distance-based, (iii) intensity threshold, and (iv) manual verification.

Machine Learning Approach

MicroLive uses a machine learning classifier to determine spot colocalization in a test image crop. This classifier is implemented as a Convolutional Neural Network (CNN) given in `microlive/ml_spot_detection.py`. The CNN architecture (See Figure S2) comprises two convolutional blocks, each followed by ReLU activation and max-pooling. The pooled feature map is flattened and passed through a fully connected layer with ReLU activation, followed by a final linear layer with sigmoid activation to yield a probability of spot presence.

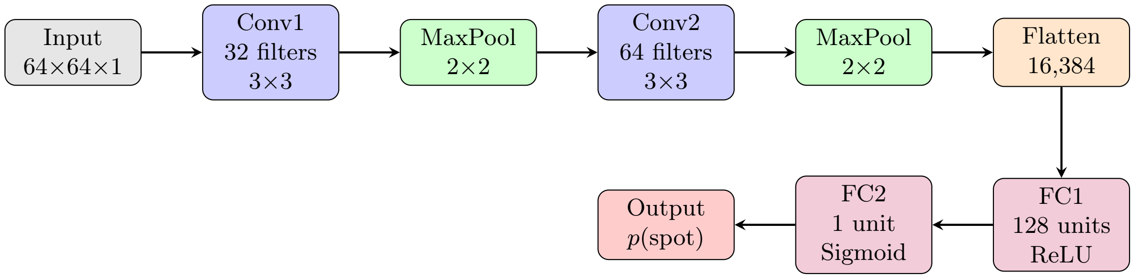

Figure S2: CNN architecture for spot detection. The network receives a 64×64 grayscale image crop and outputs a probability indicating the presence of a fluorescent spot.

Training Dataset

The final CNN model deployed in MicroLive was trained using a human-consensus labeled dataset. Three independent annotators (laboratory members with expertise in single-molecule microscopy) independently labeled image crops extracted from tracked spots in live-cell translation imaging experiments. The consensus labels were determined as follows: a crop was labeled "positive" (spot present, n =69) only if all three annotators agreed, and "negative" (no spot, n = 51) only if none of the annotators identified a spot. Ambiguous cases where annotators disagreed were excluded from the training set. The network was trained using a subset of these consensus-labeled image crops combined with data augmentation. The network was trained using the Adam optimizer with a learning rate of 10^{-6} and a batch size of 256.

Validation Against Human Annotations

To evaluate the CNN performance, we compared model predictions against the human consensus ground truth on the 120 consensus-labeled image crops. Performance was assessed by computing accuracy, precision, recall, and F1-score. The consensus-trained model achieved high accuracy at the default threshold of 0.51. Performance metrics are summarized in Table S1.

**Threshold**	**TP**	**FP**	**TN**	**FN**	**Accuracy**	**Precision**	**Recall**	**F1**
t=0.50	69	51	0	0	57.5%	57.5%	100%	0.73
t=0.51 (default)	68	2	49	1	97.5%	97.1%	98.6%	0.98
t=0.67	67	1	50	2	97.5%	98.5%	97.1%	0.98

Table S1: CNN validation metrics against human consensus labels. TP = True Positive, FP = False Positive, TN = True Negative, FN = False Negative. Precision = TP/(TP+FP); Recall = TP/(TP+FN); F1 = 2×Precision×Recall/(Precision+Recall).

At runtime, MicroLive loads the pretrained weights, and during analysis the user first selects a reference channel for spot detection using the tracking algorithms; for each detected spot, a crop from the test channel is reshaped and fed to the CNN. The output probability is compared against a user-adjustable threshold (default 0.51) to produce a binary colocalization decision. Users can adjust this threshold to optimize the trade-off between false positives and false negatives for their specific imaging conditions.

Distance-Based Approach

This method uses spatial distance thresholds to determine colocalization between tracked spots in different channels. For each tracked spot in the reference channel, the algorithm computes the Euclidean distance to all spots in the test channel at the same time frame. Spots are considered colocalized if their spatial distance falls below a user-defined threshold (in nanometers or pixels). This approach is available when multi-channel tracking has been performed independently for each channel.

Intensity Threshold Approach

This method uses a fluorescence intensity cutoff to determine whether the spots in the reference and test channels are colocalized. For each reference spot centroid, the algorithm checks whether the same coordinates (within a specified radius) in the test channel exhibit a local fluorescence intensity above the user-defined cutoff.

Manual Verification Approach

Manual verification is implemented by showing the user side-by-side crops for the reference and test channels. The user can select a checkbox to determine the presence of colocalized spots. This method can be pre-populated with the results obtained from the machine learning, distance-based, and intensity threshold approaches, allowing the user to only manually verify a subset of samples.

Correlation Analyses

MicroLive enables users to perform autocorrelation analysis on the intensity time courses of individual fluorescent spots to quantify temporal persistence and fluctuation dynamics. The autocorrelation function (ACF) of a fluorescence trace $I(t)$ is defined as

$$G(\tau) = \frac{\langle \delta I(t) \delta I(t+\tau) \rangle}{\langle I(t) \rangle^2}, \tag{10}$$

where $\delta I(t) = I(t) - \langle I(t) \rangle$ and τ is a time lag. This function measures how fluctuations in intensity at a given time t correlate with those at a later time $t + \tau$. Before computing autocorrelations, trajectories that are too short or have insufficient signal-to-noise can be filtered out to ensure reliable statistics. Additional noise-mitigation steps can be applied, for example, the autocorrelation at $\tau = 0$ (which can be affected by shot noise) can be replaced by an interpolated value from the first few non-zero lag points, any baseline offset at long τ can be subtracted so that the correlation approaches zero at large lag. The resulting ACF curves (averaged over all trajectories detected in the ROI) can be fitted with linear or exponential decay models to extract the correlation decay time τ_c. Bootstrap resampling of trajectories is used to estimate standard errors (Coulon and Larson, 2016).

Biophysical parameters were estimated as in Larson et al. (2011), that is, elongation rates were approximated as follows:

$$k_e \approx \frac{L}{\tau_c}\,, \tag{11}$$

where L is the gene length, and initiation rates were approximated as follows:

$$k_i \approx \frac{1}{G(0) \cdot \tau_c}\,, \tag{12}$$

where $G(0)$ is the value of the autocorrelation function at $\tau = 0$.

Verification Using a Synthetic Dataset

To assess the performance and accuracy of MicroLive, we compared its outputs with a known ground-truth synthetic dataset generated using the rSNAPed library, where all parameters are predetermined (Raymond et al., 2023). In short, the rSNAPed library simulates live-cell single-molecule translation movies by first importing a real cell video loaded with fluorophores as the background of the movie; then adding simulated translation spots with fluctuating fluorescence intensities calculated using a TASEP (Totally Asymmetric Exclusion Process) model, which takes into account the ribosomal initiation rates, elongation rates, and ribosomal exclusion. The intensity of each spot is proportional to the number of simulated ribosomes and their elongation rates. This simulated movie provides an approach to verify the performance and accuracy of MicroLive, as we know the true photobleaching decay rate and the true characteristics of the simulated spots, including spot size, spot intensity, number of spots, diffusion rate, and the initiation and elongation rates. We used rSNAPed to simulate the translation of smHA-KDM5B-BoxB-MS2 (1,910 codons, plasmid sequence is provided in the GitHub repository) using the parameters given in Table S2. We loaded the simulated movie into MicroLive, processed it, and calculated all parameters relevant to spot properties and dynamics. We found a strong agreement between the ground-truth dataset and MicroLive quantification (Figure S3).

**Parameter**	**rSNAPed**	MicroLive
k_e	5.0 aa/s	5.0 aa/s
k_i	0.040 s^{-1}	0.032 s^{-1}
Diffusion rate	0.100 px^2/s	0.101 px^2/s
σ_{psf}	1.50 px	1.46 px
Photobleaching decay rate	0.001 s^{-1}	0.001 s^{-1}

Table S2: Parameters used in rSNAPed for generating a simulated single-molecule translation movie of smHA-KDM5B-BoxB-MS2 construct.

Testing with a Real Microscopy Dataset

To evaluate the performance of MicroLive on real experimental data, we tested MicroLive on our acquired microscopy single-molecule translation movies of smHA-KDM5B-BoxB-MS2 (Galindo et al., 2025). First, we generated a U-2 OS cell line stably expressing both anti-HA-frankenbody-HaloTag and tdMCP-tdmSG probes as well as λN-CAAX for tethering mRNAs to the plasma membrane upon binding the BoxB stem loops in the 3' UTR, all of which are under a Tet-On promoter. The day before imaging, we transiently transfected the stable cells seeded on MatTek chambers with the smHA-KDM5B-BoxB-MS2 construct in Opti-MEM for 3h, then replaced the medium with DMEM(+) containing 1 μg/mL doxycycline and 100 nM of JF646 dye. On the imaging day, the cells were washed three times and fed with 1mL of DMEM(+) medium without phenol-red for imaging. We acquired time-lapse movies (600 frames at 1 fps, single z plane, and a pixel size of 130 nm) on our Leica Stellaris 5 confocal microscope using a 63x oil immersion objective, 2% laser power at 488 nm (RNA channel) and 5% at 638 nm (nascent chain channel). The LIF files were imported into MicroLive for cell segmentation, particle tracking, spot intensity extraction, and autocorrelation analysis. MicroLive reliably detected spots and linked trajectories longer than 25 frames, yielding a mean decorrelation time of $\approx$ 100 seconds (Figure S4).

Comparison with Existing Tools

Several software tools are commonly used for fluorescence microscopy image analysis, but each has limitations for live-cell single-molecule imaging workflows. ImageJ/Fiji (Schindelin et al., 2012) offers flexibility through its plugin ecosystem, but achieving a complete live-cell imaging pipeline requires installing multiple plugins and writing custom macros to connect them. FISH-quant (Imbert et al., 2022) has been developed for fixed-cell smFISH spot detection but lacks native support for time-lapse analysis, trajectory linking, and

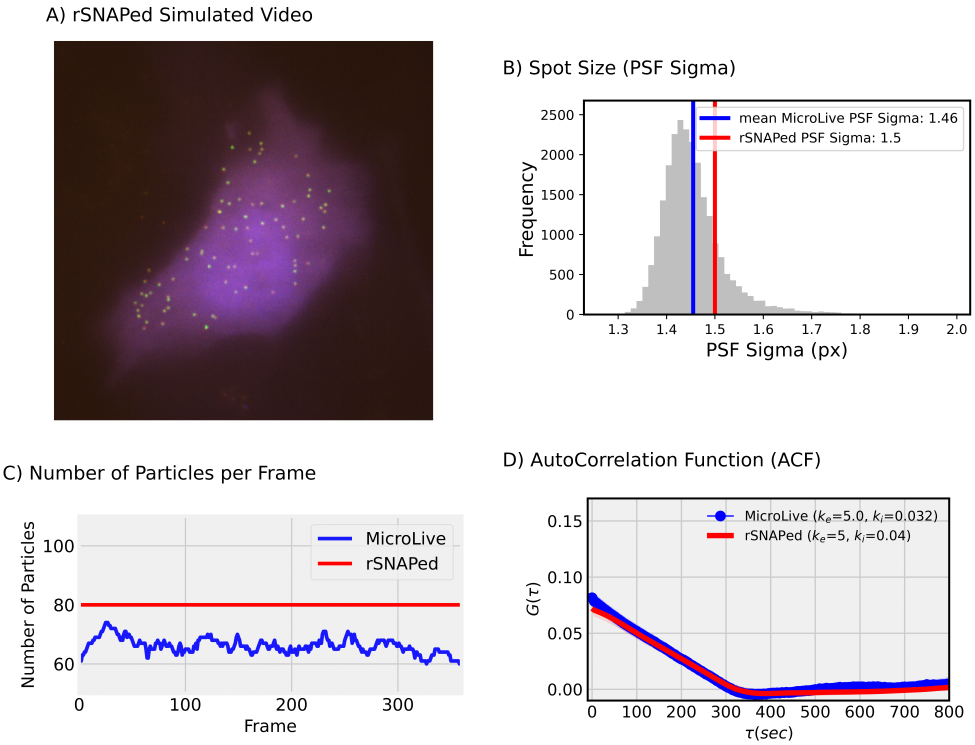

Figure S3: MicroLive image processing results for a synthetic image generated by the rSNAPed library. A) The top image shows a simulated microscopy image, representing the translation of the smHA-KDM5B-BoxB-MS2 construct using the rSNAPed library and the parameters provided in Table S2. B) Comparison of the spot size used to generate the simulated dataset (red line) with the quantified spot size by MicroLive (blue line). C) Time course showing the number of detected trajectories longer than 25 frames. 2D-tracking was performed using a particle size of 5 pixels and a search range of 7 pixels. The red line indicates the number of particles used for the simulation. D) Autocorrelation function calculated using the simulated data from rSNAPed (red line), and autocorrelation function calculated using the intensity recovered from tracked spots with MicroLive (blue line).

photobleaching correction. Custom Mathematica or Matlab scripts (Lyon et al., 2019; Khuperkar et al., 2020) provide flexibility but require programming expertise, are commonly developed independently by each laboratory, and depend on proprietary software that requires a paid license. In contrast, MicroLive integrates all major analysis steps within a single graphical interface, including segmentation, 2D/3D particle detection, photobleaching correction, trajectory linking, autocorrelation analysis, and CNN-based colocalization. By consolidating these capabilities into an accessible open-source platform with automatic metadata export, MicroLive eliminates the need to chain together independent tools or develop custom code, lowering the barrier for quantitative live-cell imaging.

Documentation

Technical and user documentation for MicroLive is provided in its GitHub repository `https://github.com/ningzhaoAnschutz/microlive` in the following links:

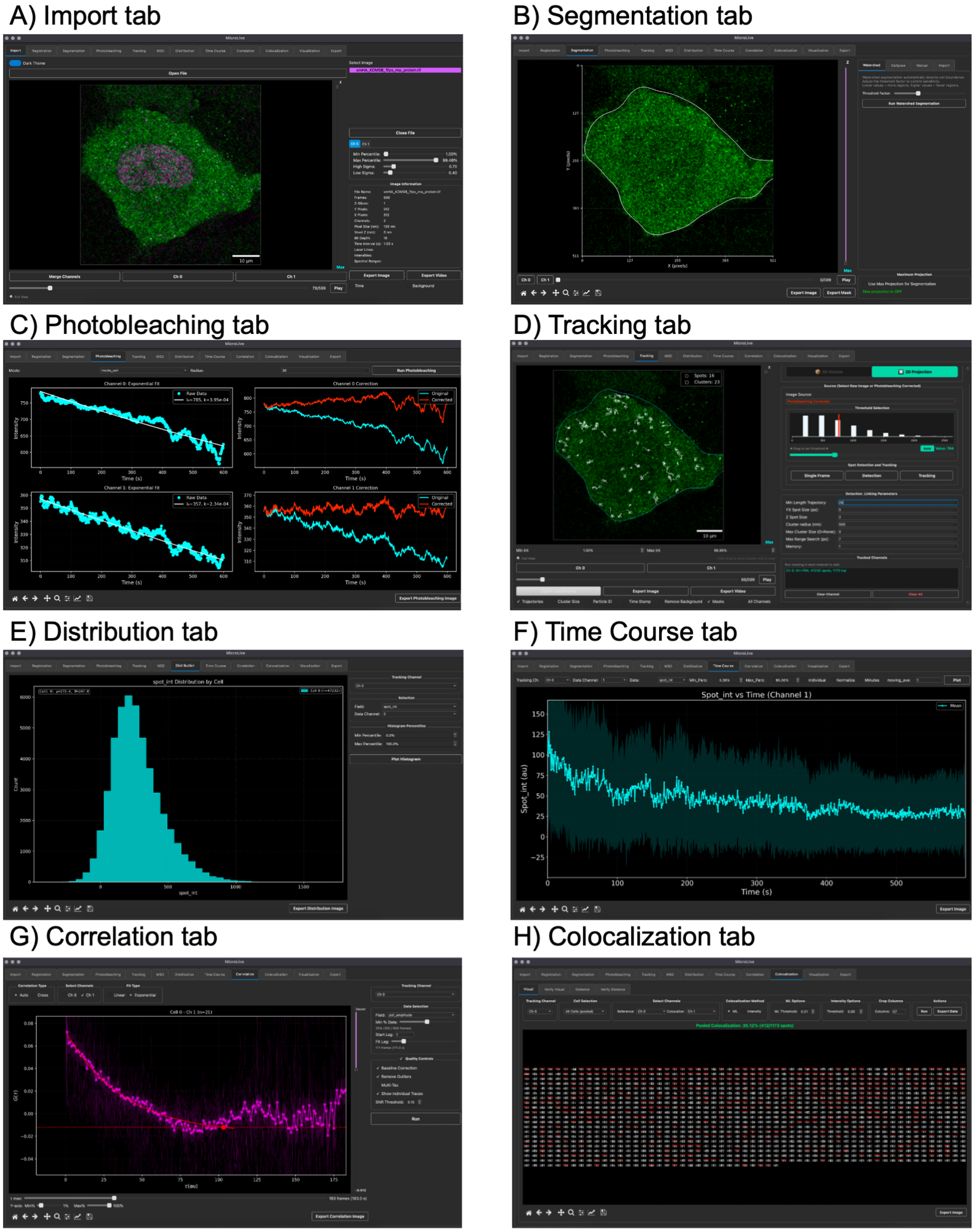

Figure S4: Screenshots show the main tabs in MicroLive. A) Import tab, showing a representative microscopy movie where the green channel represents the mRNA channel, and the magenta channel represents the nascent chain channel. B) Segmentation tab. C) Photobleaching tab. D) Representative image showing the particle tracking results at a given time frame. E) Distribution tab showing the spot intensity for all spots detected in all frames. F) Time course tab showing the particle intensity over time. G) Correlation tab. A representative plot showing the autocorrelation function of intensity calculated from the trajectories of the nascent chains. H) Colocalization tab, showing pairs of crops for the mRNA channel and the protein channel. The red bar at the top indicates where the code is detecting a pair of colocalized spots.

**Document**	**GitHub Link**
Complete user manual	user_guide.md
Step-by-step tutorials	tutorial.md
Technical API documentation	api_reference.md

References

Allan, D. B., Caswell, T., Keim, N. C., van der Wel, C. M., and Verweij, R. W. (2025). Soft-matter/trackpy: V0.7. Zenodo.

Coulon, A. and Larson, D. R. (2016). Fluctuation analysis: dissecting transcriptional kinetics with signal theory. In *Methods in Enzymology*, volume 572, pages 159–191.

Galindo, G., Fixen, G. M., Heredia, A., Morisaki, T., and Stasevich, T. J. (2025). All probes plasmids (APPs) for multicolor and long-term tracking of single-mRNA translation dynamics. *Molecular Biology of the Cell*, 36(6):mr6.

Hospelhorn, B. G., Kesler, B. K., Jashnsaz, H., and Neuert, G. (2025). TrueSpot: a robust automated tool for quantifying signal puncta in fluorescent imaging. *Genome Biology*, 26:317.

Imbert, A., Ouyang, W., Safieddine, A., Coleno, E., Zimmer, C., Bertrand, E., Walter, T., and Mueller, F. (2022). FISH-quant v2: a scalable and modular tool for smFISH image analysis. *RNA*, 28(6):786–795.

Khuperkar, D., Hoek, T. A., Sonneveld, S., Verhagen, B. M. P., Boersma, S., and Tanenbaum, M. E. (2020). Quantification of mRNA translation in live cells using single-molecule imaging. *Nature Protocols*, 15(4):1371–1398.

Larson, D. R., Zenklusen, D., Wu, B., Chao, J. A., and Singer, R. H. (2011). Real-time observation of transcription initiation and elongation on an endogenous yeast gene. *Science*, 332(6028):475–478.

Lyon, K., Aguilera, L. U., Morisaki, T., Munsky, B., and Stasevich, T. J. (2019). Live-cell single RNA imaging reveals bursts of translational frameshifting. *Molecular Cell*, 75(1):172–183.

Miura, K. (2020). Bleach correction ImageJ plugin for compensating the photobleaching of time-lapse sequences. *F1000Research*, 9:1494.

Raymond, W. S., Ghaffari, S., Aguilera, L. U., Ron, E., Morisaki, T., Fox, Z. R., May, M. P., Stasevich, T. J., and Munsky, B. (2023). Using mechanistic models and machine learning to design single-color multiplexed nascent chain tracking experiments. *Frontiers in Cell and Developmental Biology*, 11:1151318.

Schindelin, J., Arganda-Carreras, I., Frise, E., Kaynig, V., Longair, M., Pietzsch, T., Preibisch, S., Rueden, C., Saalfeld, S., Schmid, B., Tinevez, J.-Y., White, D. J., Hartenstein, V., Eliceiri, K., Tomancak, P., and Cardona, A. (2012). Fiji: an open-source platform for biological-image analysis. *Nature Methods*, 9(7):676–682.

Stringer, C., Wang, T., Michaelos, M., and Pachitariu, M. (2021). Cellpose: a generalist algorithm for cellular segmentation. *Nature Methods*, 18(1):100–106.

Vincent, L. and Soille, P. (1991). Watersheds in digital spaces: an efficient algorithm based on immersion simulations. *IEEE Transactions on Pattern Analysis & Machine Intelligence*, 13(6):583–598.
